# Circadian Strain, Light Exposure, and Depressive Symptoms in Rural Communities of Southern Brazil 

**DOI:** 10.3389/fnetp.2021.779136

**Published:** 2022-01-26

**Authors:** Luísa K. Pilz, Nicóli B. Xavier, Rosa Levandovski, Melissa A. B. Oliveira, André C. Tonon, Débora B. Constantino, Valdomiro Machado, Till Roenneberg, Maria Paz Hidalgo

**Affiliations:** ^1^ Laboratório de Cronobiologia e Sono, HCPA/ UFRGS, Porto Alegre, Brazil; ^2^ Graduate Program in Psychiatry and Behavioral Sciences, UFRGS, Porto Alegre, Brazil; ^3^ Institute of Medical Psychology, LMU Munich, München, Germany; ^4^ PPG Avaliação e Produção de Tecnologias para o SUS, GHC, PPG Saúde Coletiva, UFRGS, Porto Alegre, Brazil

**Keywords:** actigraphy, mood, circadian rhythm, lighting, depression

## Abstract

Irregular light–dark cycles and circadian/sleep disturbances have been suggested as risk or co-occurring factors in depression. Among a set of metrics developed to quantify strain on the circadian system, social jetlag (SJL) has been put forward as a measure of the discrepancy between biological and social clocks. Here, we approached the question on whether light exposure and SJL would also be associated with depressive symptoms in Quilombola communities in Southern Brazil. These rural communities are void of potential confounders of modern lifestyles and show low levels of SJL. 210 Quilombolas (age range 16–92; 56% women) were asked about their sleep times and light exposure using the Munich ChronoType Questionnaire (MCTQ). The Beck Depression Inventory (BDI) was used to assess depressive symptoms. Additionally, we analyzed 7-day actimetry recordings in 124 subjects. BDI scores higher than 10 (having clinically significant depressive symptoms; controlled for age and sex in the multivariate analysis) were positively associated with SJL >1 h and negatively associated with median light exposure during the day, especially in the morning from 8:00 to 10:00. Our results suggest that low light exposure during the day, and higher levels of SJL are associated with depressive symptoms; longitudinal and experimental studies are needed to understand the underlying mechanisms. Nevertheless, we highlight the potential of treatment strategies aimed at decreasing circadian strain and insufficient light exposure, which are suggested as areas of further research in Psychiatry.

## Introduction

The increasing prevalence of mood disorders and the suboptimal efficacy of antidepressants are related to our limited understanding of risk factors and underlying pathophysiology. Irregular light–dark cycles and circadian/sleep disturbances have been suggested as risk or co-occurring factors in depression (Bedrosian and Nelson, 2017). Studies with large sample sizes suggest negative associations between working outdoors and depressive symptoms (Hahn et al., 2011; Asai et al., 2018); naturalistic studies have shown associations between light exposure profiles (i.e., decreased light exposure during the day and/or increased light exposure at night) and depression or depressive symptoms severity (Haynes et al., 2005; Park et al., 2007; Endo et al., 2014; Paksarian et al., 2020). In addition, there are positive short-term effects of bright light exposure on mood (aan het Rot et al., 2008).

Light–dark cycles are the most important synchronizing signal (*zeitgeber*) that entrain our clocks to the 24-h day (Minors et al., 1991; Roenneberg and Merrow, 2005). Of note, body clocks orchestrate the daily timing of physiology, from gene expression to behavior. In industrial societies, under weak *zeitgebers*, most body clocks delay, but work times have not adapted to these changes. As a result, biological and social time drifted apart (Roenneberg and Merrow, 2005).

Mood disorders are also often accompanied by disruption of circadian rhythms, and although causal relationships are still not clear, overlapping phenomena and mechanisms have been documented (Bechtel, 2015). Part of this knowledge was gathered from studying the health of shift workers, who are mostly under conditions of great circadian disruption. A recent meta-analysis of prospective studies showed that the risk of depressive symptoms was 33% higher among them (Torquati et al., 2019).

Even exposure to relatively weaker challenges (i.e., than shift work) may result in significant disruption of the temporal order of the organisms (Beauvalet et al., 2017)**.** Among a set of metrics developed to quantify strain on the circadian system (henceforth referred to as *circadian strain*), social jetlag (SJL) has been put forward as a measure of the discrepancy between biological and social clocks (Wittmann et al., 2006). SJL, like shift work, is mainly a consequence of occupational schedules, but even more prevalent (Roenneberg et al., 2019). SJL simply quantifies the difference between sleep timing on work and work-free days. Some studies showed an association of SJL with depressive symptoms (Levandovski et al., 2011; Islam et al., 2020), while a study with clinical samples did not (Knapen et al., 2018). Other metrics for circadian strain, such as instability/fragmentation (Luik et al., 2013) or amplitude (Hori et al., 2016) of activity rhythms, as recorded by actimetry, were also associated with depressive symptoms, although no differences in inter-daily stability were seen in depressed patients compared with controls (Berle et al., 2010). Inconsistent findings in the literature might be a consequence of methodological heterogeneity and of the diverse characteristics of each sample, especially with a multifactorial disorder such as depression.

Here, we report results from analyzing the relationships between circadian strain, light exposure, and depressive symptoms in Quilombola communities. Quilombolas often live in settlements (Quilombos) originally founded in remote areas, far from large urban centers. These communities are known as remaining social groups established in the past to escape or resist slavery (and slavery remnants) in Brazil with an established ethnic identity and culture. The sociocultural background is relatively uniform throughout these communities. They are present across Brazil and genetic surveys so far have suggested a pattern of admixture among Africans, Europeans, and Native Americans in different proportions (Scliar et al., 2009; Ribeiro et al., 2011; Kimura et al., 2013; Gontijo et al., 2014; Kimura et al., 2017). Those selected for this study are located in rural areas in the south of Brazil and have different histories of access to electricity (Pilz et al., 2018). In contrast to the industrialized urban 24/7 life-style, work activities in many of these rural Quilombos, are very much tied to “solar time,” resulting in low levels of SJL. Thus, they are a unique population for studying the effects of light on daily behavior and mood without the confounders of modern life-styles (i.e., patterns of light exposure and SJL may also be associated with other risk factors for depression, such as social inequality and household structure). We hypothesized that low light exposure during the day, SJL, and other circadian strain measures (derived from rest-activity patterns) would be associated with depressive symptoms in this population.

## Materials and methods

### Participants and recruitment process

We included 210 participants (73% of the 287 recruited, with no significant difference in age, sex, or BDI between subjects included vs. excluded; see Supplementary Figure S1 for exclusion criteria) from rural Quilombos, seven of which have been characterized in a previous study (Pilz et al., 2018). These individuals are all Portuguese native speakers, older than 16 years of age, living in 12 different communities across four states in the South of Brazil with similar latitudes and cultural background (Supplementary Figure S2). All data were collected from March 2012 to March 2017 (for sample characterization, see Table 1; for a description of occupations in the sample, see Supplementary Table S2).

**TABLE 1:** Sample characteristics.

	MCTQ (N = 210)	Actimetry (N = 124)	Skin temperature (N = 58)
Age: median (Q_1_—Q_3_)	47 (31–58)	48 (31–60)	36 (22–50)
Sex, female: n (%)	118 (56%)	72 (58%)	30 (52%)
Reported antidepressants medication usage: n (%) (Fluoxetine/Amitriptyline)	8 (4%)	9 (7%)	4 (7%)
**Schooling: n (%)**			
Illiterate	31 (15%)	21 (17%)	6 (10%)
Primary School incomplete (1st—4th grade)	87 (41%)	53 (43%)	30 (52%)
Primary School incomplete (5th—7th grade)	32 (15%)	21 (17%)	9 (16%)
Primary School complete	16 (8%)	6 (5%)	1 (2%)
High School incomplete	11 (5%)	6 (5%)	5 (9%)
High School complete	11 (5%)	7 (5.5%)	3 (5%)
Undergraduate incomplete/graduate degree	4 (2%)	2 (1.5%)	2 (3%)
Not reported	18 (9%)	8 (6%)	2 (3%)
**Beck Depression Inventory (BDI) score**:			
median (Q_1_—Q_3_)^a^	4 (2–9)	5 (3–9)	5 (3–9)
BDI >10 (clinically significant symptoms): n (%)	47 (22%)	29 (23%)	12 (21%)
**MCTQ variables**: median (Q_1_—Q_3_)			
Midsleep on workdays (MSW; hh:mm)	2:32 (1:50–3:14)	2:19 (1:35–3:06)	1:45 (1:30–2:19)
Midsleep on work-free days (MSF; hh:mm)	3:00 (2:11–4:00)	2:45 (2:00–3:41)	2:30 (1:35–3:15)
Sleep duration on workdays (h)	7.7 (6.8–8.8)	7.7 (7.0–8.8)	8.4 (7.3–9.0)
Sleep duration on work-free days (h)	8.3 (7.1–9.2)	8.5 (7.2–9.5)	8.9 (7.9–10.0)
Time spent outdoors on workdays (h)^b^	7.0 (3.3–10.0)	7.0 (3.1–9.0)	6.7 (4.0–8.0)
Time spent outdoors on work-free days (h)	5.0 (2.0–8.9)	5.0 (2.5–8.0)	4.0 (2.0–7.0)
Social jetlag (SJL; h)	0.25 (0.0–1.00)	0.0 (0.0–0.89)	0.2 (0.0–1.0)

Note. Schooling: primary school, “Ensino Fundamental” time from first to eighth grade in Brazil.

a In the MCTQ sample, 16 individuals did not complete the questionnaire but could still be categorized as having or not clinically significant symptoms (BDI >10); nine in the actimetry sample and two in the temperature. Therefore, BDI, median refers to N = 194/115/56.

b In the MCTQ, sample, 3 individuals could not estimate time spent outdoors on workdays; in the actimetry sample, 14 and 12 (activity/light and temperature).

BDI, Beck depression inventory; MCTQ, Munich ChronoType Questionnaire.

All participants signed a written informed consent. Parents also gave their consent for participants younger than 18. Informed consent for illiterate participants was obtained in the presence of a literate Quilombola witness. Procedures were carried out in accordance with the Declaration of Helsinki, and approved by the Ethics Committee of the Hospital de Clínicas de Porto Alegre (#2011–0502 and #2015–0568). According to ethical recommendations, the research team first contacted the community leaders as is the customs of the Quilombolas. Local meetings were held in the community center of the respective Quilombo. On these occasions, our team would inform the Quilombolas about the study and invite them to participate. Data collection was conducted either at the headquarters or at the homes of the participants.

### Materials

#### Questionnaires

Team members were trained to perform standardized interviews about sleep-wake behavior and average natural light exposure (Munich ChronoType Questionnaire; MCTQ), as well as depressive symptoms (Beck Depression Inventory; BDI). Whenever necessary, questions were adapted to the Quilombolas cultural context (e.g., using synonyms to facilitate comprehension). Demographic characteristics were collected using a standard questionnaire (Carvalho et al., 2014).

##### Munich ChronoType Questionnaire (MCTQ)

The MCTQ (Roenneberg et al., 2003) is an instrument that probes sleep–wake behavior and natural light exposure separately for workdays and work-free days. From MCTQ data, we computed midpoint of sleep on free days (as a marker of phase of entrainment; MSF), social jetlag (as an estimate of circadian strain; SJL), and the amount of time spent outdoors on free days (as a proxy for natural light exposure). We used data from free days, as those usually reflect circadian states less restricted by work/social constraints. Further information on the calculation of the variables and the full English version of the instrument can be assessed at http://thewep.org/documentations/mctq.

##### Beck Depression Inventory (BDI)

The BDI assesses symptoms and behaviors related to depressive symptomatology (Beck et al., 1961; Beck et al., 1996). In this study, we used the Beck Depression Inventory-I and chose a cut-off score of more than 10 to describe clinically significant depressive symptoms. This value was chosen according to previously published data for non-clinical populations (Gomes-Oliveira et al., 2012) and is similar to the cut-off suggested by Beck et al. (1988) for clinical samples (≥10). When categories were used for analyses, and the questionnaire was incomplete due to one single item, participants were still included if they could be categorized (scores below 8 or above 10). A Brazilian Portuguese validated version was used (Gorenstein and Andrade, 1996).

#### Actimetry

Actimeters are wrist-worn devices that measure locomotor activity by accelerometry and, additionally, record light and skin temperature information. From the total sample size (n = 287), 171 participants received wrist actimeters (Actiwatch-2: Philips Respironics, ActTrust: Condor) for continuous activity data collection. The devices used were previously shown not to differ in sleep detection (Pilz et al., 2018). Data from 124 (66 Actiwatch-2, 58 ActTrust) subjects could be used. Actimetry analyses were conducted once inclusion criteria were satisfied: continuous actimeter use for at least 7 (n = 124) or 14 days (n = 100) with no more than 4 h of missing data (per day). Parameters were computed using 7/14 days, in order to have one of each weekday included for every participant.

Data were binned into 10 min for analyses. To make the data from both actimeters comparable, light recordings were normalized using the correlation slope equation from the data collected over 14 days using both actimeters at the same time as previously described (Pilz et al., 2018). Activity data from ActTrust were normalized according to the recommendations of the manufacturers to be comparable with Actiwatch 2. Skin temperature data were only available when ActTrust was used (58 subjects, 7 days; 46 subjects, 14 days). The only significant difference in age/sex/BDI we detected between the participants included in the actimetry analysis (124—light/activity and 58—temperature) vs. those participants recruited but excluded was a lower age in the temperature set. The participants in the temperature dataset also have a lower age as compared with the MCTQ and activity/light datasets.

Missing data (actimeter considered off-wrist) were detected as stretches of zero of at least 10 consecutive 10-min bins and confirmed by visual inspection. When a day had more than 4 h of missing, it was not included. Entire days were only removed from 11 subjects in the 14-day sample (11 out of 1,400 days).

##### Parameters derived from actimeters (continuous recordings)

The onset of time series was set at 0:00 for all variables. We computed individual profiles using the median activity/temperature/light across time of day of each subject. The median of individual profiles was used to plot the daily profiles of the group according to the presence or not of clinically significant depressive symptoms. The time intervals, at which we compared median light exposure, were chosen based on our visual inspection of grouped profiles and photoperiod in the south of Brazil.

We also computed each subject's activity and skin temperature rhythms': 1) inter-daily stability (IS), indicating how stable the 24 h pattern was throughout the recorded days; 2) intra-daily variability (IV), which gives an estimation of rhythm fragmentation and reflects the frequency and extent of transitions between rest and activity (van Someren et al., 1996); 3) cosinor-based acrophase, a measure of when high values recur in each cycle (Halberg et al., 1967); and 4) cosinor-based amplitude, that reflects the extent of variation within a cycle. Acrophase was computed with linear fitting using the R package “*psych*” (Revelle, 2019), and amplitude was computed using the R package “*cosinor2*” (Mutak, 2018).

### Data analysis

We used Cronbach’s alpha to test the internal consistency of the BDI in Quilombolas and item-total corrected correlations to see which symptoms were more strongly correlated to the total score. Additionally, we plotted the distributions of each item scores separately by group: BDI ≤10 and BDI >10. Shapiro-Wilk and inspection of histograms were used to test for normality. Initially, we compared MSF and SJL between BDI groups using bivariate tests (Wilcoxon-Mann-Whitney, Chi-square). Then, we tested the association between MCTQ variables (MSF, SJL, and hours spent outdoors on free days) and the presence of clinically significant depressive symptoms (BDI >10), controlling for age and sex using robust (estimator HC0) Poisson regression. Robust estimators were used, since models were under-dispersed (Cummings, 2019). SJL was categorized considering the high prevalence of zeros and the regression assumption of linearity. “Hours spent outdoors on free daysˮ was also tested in the models as a categorical variable (Q_1_: ≥2 h, <2 h), without substantially changing them (data not shown).

Variables derived from actimeters data (IS, IV, acrophase, amplitude, median light exposure, and activity during the day—from 7:00 to 17:00, in the morning—from 8:00 to 10:00, and at night—from 20:00 to 1:00) were compared between individuals with (BDI >10) and without (BDI scores ≤10) clinically significant depressive symptoms using the Wilcoxon–Mann–Whitney test.

Based on bivariate analyses results and our hypotheses, we selected parameters derived from actimeters continuous recordings to test as factors in a Poisson regression model (with robust estimators) comparable to the one with MCTQ variables: light exposure from 8:00 to 10:00, acrophase (as a phase marker) and activity-IS (as a measurement of stability), which should reflect circadian strain when low values are found. Models were additionally controlled for season of data collection.

Tests were run using R packages: “*dplyr*” (Wickham et al., 2019)*,* “*ggplot2*” (Wickham et al., 2016)*, “jtools”* (Long, 2019)], and SPSS 25.

## Results

### Beck depression inventory in Quilombolas

Cronbach’s alpha of the BDI in our sample was 0.83. Item-total adjusted correlation coefficients are shown in Supplementary Table S2. Except for items “weight loss” and “suicidal thoughts/wishes,” all items had coefficients higher than 0.2. Items “sadness,” “pessimism,” “guilty feelings,” “self-dislike,” “self-criticalness” had the highest coefficients (higher than 0.5). Sum-scores ranked higher in women than in men (Supplementary Table S3). Supplementary Figure S3 shows the distribution of item scores by group (BDI ≤10 vs. BDI >10).

### Beck depression inventory scores vs. Munich ChronoType questionnaire variables

**FIGURE 1:**
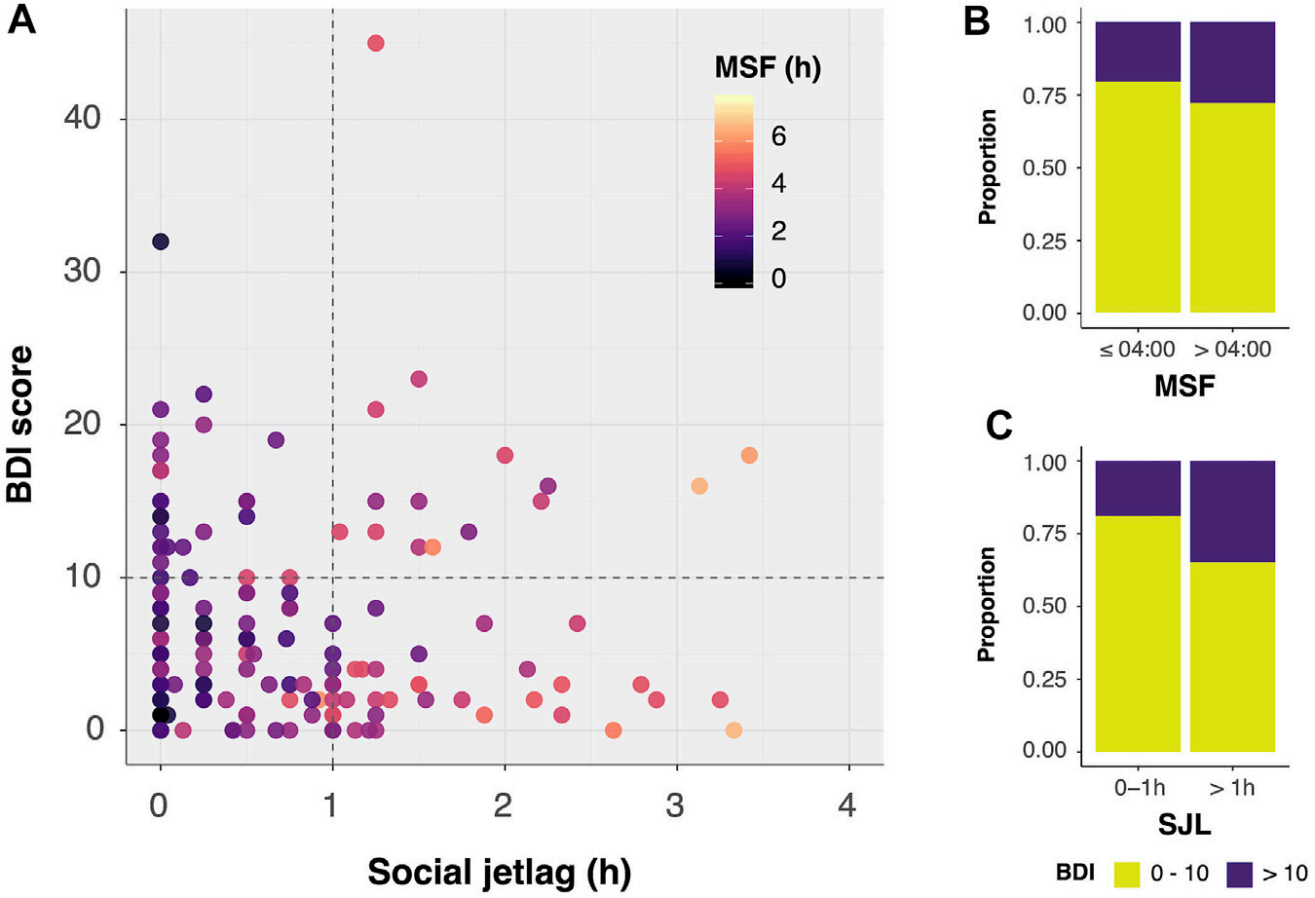
Association between Beck depression inventory (BDI) scores and social jetlag (SJL)/midpoint of sleep on free days (MSF). **(A)** Dots are color coded according to MSF: the later the MSF, the lighter the dot. BDI scores higher than 10 are significantly associated with SJL >1 h **(B)** but not with MSF later than 4:00 **(C)** Chi-square, *p < 0.05. n = 210. SJL and MSF are expressed in hours.

Although SJL was not significantly different between groups (Wilcoxon–Mann–Whitney, U = 3,568, *p* = 0.45), having a SJL of >1 h (third quartile) was significantly associated with clinically relevant symptoms (*BDI 0–10:* SJL >1 h in 18% of subjects; BDI >10*:* SJL >1 h in 34% of subjects; χ^2^ = 4.34, *p* < 0.05, Figure 1). The same is not true for having a MSF later than 4:00 (third quartile; χ^2^ = 0.81, *p* = 0.37). Table 2 shows results from Poisson regressions with having clinically significant symptoms (BDI >10) as the dependent variable. Time spent outdoors on work-free days was not significantly associated with BDI >10, whereas age was only significantly associated with it when other factors were added to the model (Models 3 and 4). MSF was significantly associated with having a BDI >10, provided SJL was not in the model. In the final model, SJL > 1 h, being a woman and older age were associated with clinically significant depressive symptoms. Supplementary Figure S4 shows SJL by BDI-group within age and sex categories.

**TABLE 2:** Poisson regression: MCTQ variables associated with BDI >10.

	Model 1	Model 2	Model 3	Model 4
(Intercept)	−2.59 (0.41)**	−2.53 (0.43)**	−3.17 (0.44)**	−3.39 (0.48)**
Age (years old)	1.01	1.01	1.01*	1.02**
	(0.99–1.02)	(0.99–1.03)	(1.00–1.03)	(1.00–1.03)
Sex (female)	2.52**	2.46**	2.33**	2.53**
	(1.36–4.68)	(1.33–4.59)	(1.25–4.34)	(1.37–4.68)
Time outdoors FD (h)	-	0.99	0.99	0.99
		(0.91–1.06)	(0.91–1.06)	(0.92–1.07)
MSF	-	-	1.19*	1.09
			(1.04–1.37)	(0.92–1.29)
Social jetlag >1 h	-	-	-	2.19**
				(1.28–3.75)
N	210	210	210	210
χ^2^	10.08**	10.21*	13.29*	17.66**
AIC	230.63	232.51	231.43	229.05
BIC	240.67	245.90	248.16	249.14
Pseudo *R* ^2^	0.07	0.07	0.09	0.12

Note. Unstandardized, exponentiated (except for intercept) coefficients and 95% confidence interval. Standard errors are heteroskedasticity robust (HC0). Pseudo *R* ^2^—Cragg–Uhler. **p < 0.01, *p < 0.05. Time outdoors FD: reported time (in hours) spent outdoors on work-free days (from MCTQ). MSF: midpoint of sleep on free days (sleep timing).

### Variables derived from actimeters data: 7- vs. 14 day-recordings

Bland–Altman plots show that estimates of IV, IS, acrophase, and amplitude of activity and temperature using 7 and 14 days were similar (Supplementary Figure S5). Correlation between variables (7 days vs. 14 days) is significant, with coefficients higher than 0.85 for all variables (Spearman). The same holds true for median light exposure estimates (Supplementary Figure S6).

### Beck depression inventory scores vs. variables computed from actimeters continuous recordings

**FIGURE 2:**
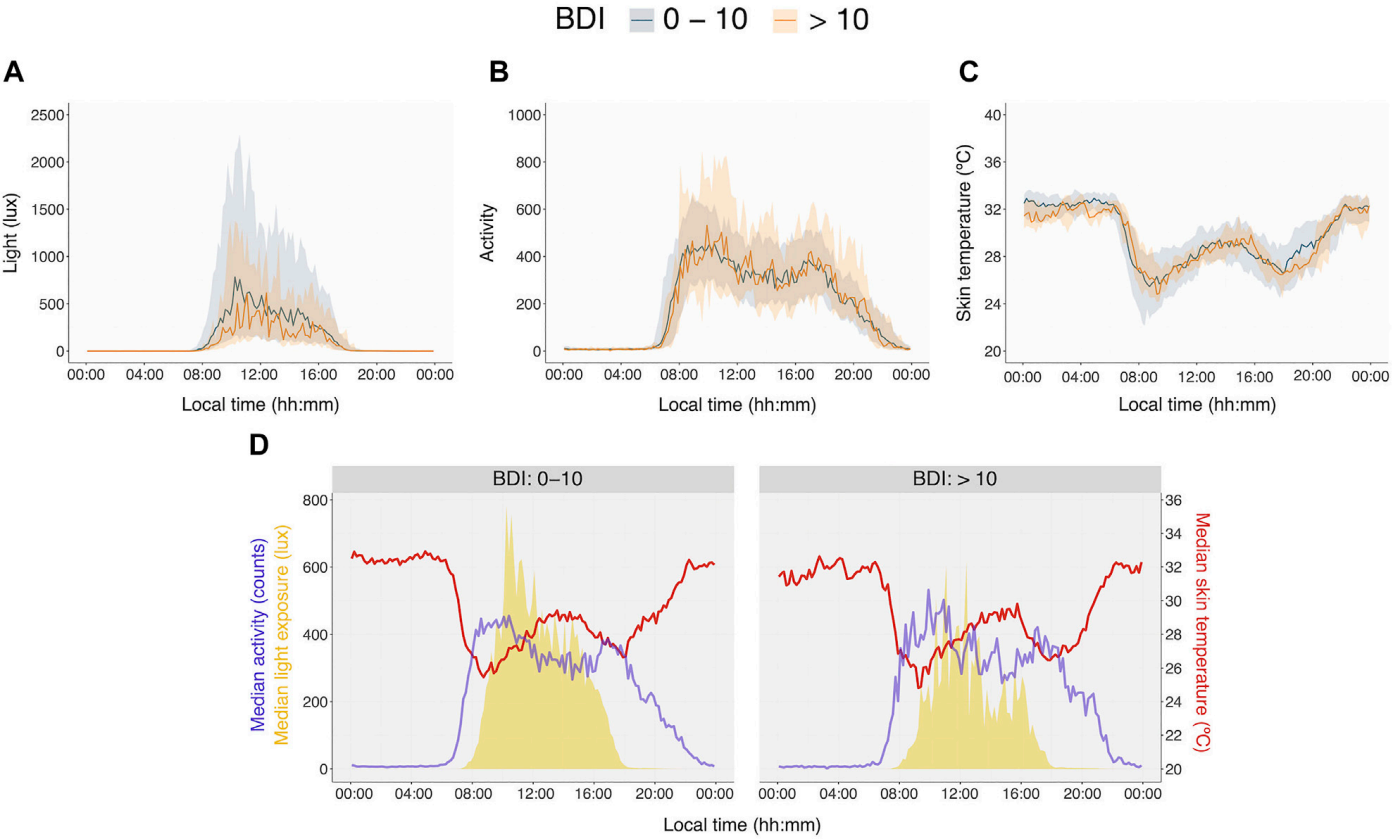
Light **(A)**, activity **(B)**, and skin temperature **(C)** group profiles of subjects with (orange) and without (blue) clinically significant depressive symptoms. Group profiles were computed using individual median profiles of 7 days. Lines represent median group profiles and the shaded area, the interquartile range [Q_1_-Q_3_]. The bottom panel **(D)** shows the same three profiles overlaid. N = 124 (activity/light). N = 58 (temperature).

Figures 2A–C show group median profiles of light exposure, activity, and temperature, respectively, as measured by actimeters in subjects with and without clinically significant depressive symptoms (BDI>10). Profiles using average instead of median and 14 days data can be seen in Supplementary Figures S7, 8, respectively.

**FIGURE 3:**
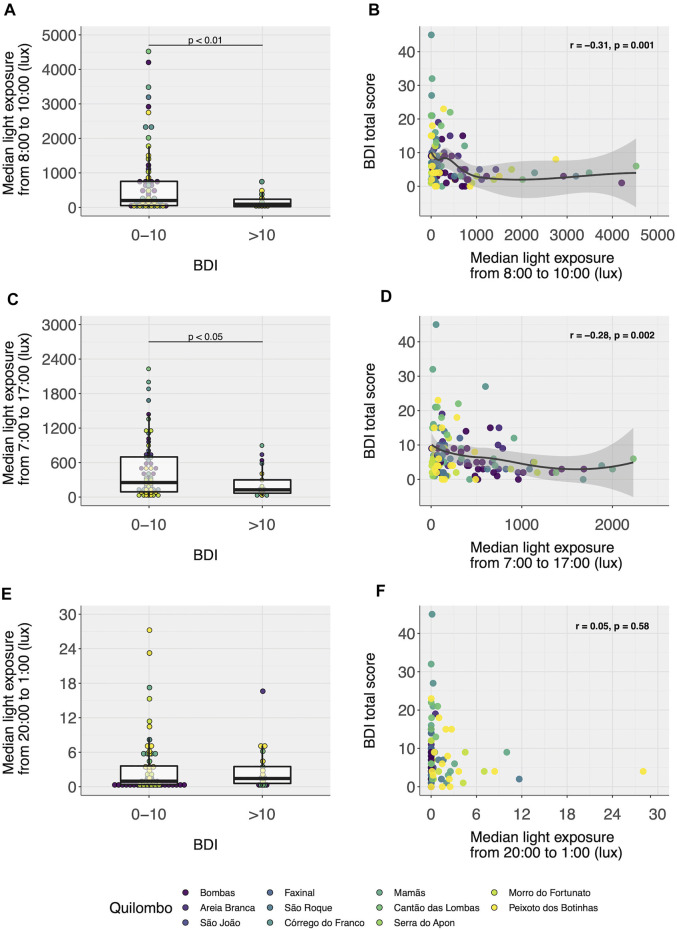
Light exposure in the morning (from 8:00 to 10:00), during the day (from 7:00 to 17:00), and at night (from 20:00 to 1:00) according to BDI scores (7 days recordings/subject). Subjects with clinically significant depressive symptoms are less exposed to light in the morning **(A)** and during the day **(C)**, but not at night **(E)**. BDI scores are also negatively correlated to median light exposure in the morning **(B)** and during the day **(D)**, but not at night **(F)**. Wilcoxon–Mann–Whitney test/Spearman’s correlation. N = 124.

Only light exposure during the day (from 7:00 to 17:00) was significantly lower (Wilcoxon–Mann–Whitney, U = 1744, *p* < 0.05) in individuals with BDI scores >10, especially in the morning between 8:00 and 10:00 (see Figure 3A; U = 1,829, *p* < 0.01) while light exposure in the afternoon, between 13:00 to 15:00, was at the significance threshold (U = 1,708, *p* = 0.05) and light exposure at night from 20:00 to 1:00 was not significantly different between groups (see Figure 3E; U = 1,273, *p* = 0.54). BDI scores correlated significantly with light exposure during the day (Figure 3). Similar results were seen using recordings of 14 days (Supplementary Figure S8 for 14 days-profiles and **S9** for group comparisons using 14 days light-recordings).

No difference was found between groups regarding levels of activity during the day or at night. The associations between BDI scores and light medians were not extended to activity IS/IV either (Supplementary Figure S10). Despite not detecting differences between groups in temperature IS with 7 days of recordings (Wilcoxon–Mann–Whitney, U = 359.5, *p* = 0.11; Supplementary Figure S10), when using data of 14 days, we found IS of temperature to be significantly lower in the BDI >10 group (U = 255.5, *p* < 0.05; Supplementary Figure S11E).

Table 3 shows the multivariate analysis results. Considering the smaller sample sizes, we started with the full model (model 1) and removed tested factors that were not significantly associated with the outcome (model 2) for parsimony. Lower median light exposure in the morning was significantly associated with having clinically relevant symptoms (from 8:00 to 10:00, unity represents an increase of 500 lux). Supplementary Figure S12 shows median light exposure in the morning by BDI-group within age and sex categories.

**TABLE 3:** Poisson regression—actimetry variables associated with BDI >10.

	Model 1 actimetry–7 days	Model 2 actimetry–7 days	Model 1 actimetry–14 days	Model 2 actimetry–14 days
(Intercept)	−1.10 (2.75)	−1.56 (0.54) **	0.49 (3.26)	−1.28 (0.62)*
Age	1.01	1.01	1.00	1.00
	(0.99–1.02)	(0.99–1.02)	(0.99–1.02)	(0.99–1.02)
Sex (female)	1.27	1.31	1.29	1.43
	(0.58–2.76)	(0.63–2.73)	(0.56–2.97)	(0.63–3.24)
Season of data collection^b^ (Mar. 20—Sep. 22)	0.76	0.86	0.58	0.79
	(0.38–1.52)	(0.46–1.60)	(0.25–1.36)	(0.39–1.63)
Median light exposure from 8:00 to 10:00 (500 lux)^a^	0.44**	0.48**	0.12**	0.19**
	(0.25–0.79)	(0.28–0.81)	(0.03–0.51)	(0.06–0.63)
Activity acrophase	0.95	-	0.86	-
	(0.69–1.31)		(0.57–1.28)	
Interdaily stability (IS: 0–100)	1.01	-	1.02	-
	(0.98–1.04)		(0.98–1.06)	
N	124	124	100	100
χ^2^	11.34	10.96*	13.42*	12.09*
AIC	144.93	141.31	111.19	108.53
BIC	164.67	155.41	129.43	121.56
Pseudo *R* ^2^	0.13	0.12	0.19	0.17

Note. Unstandardized, exponentiated (except for intercept) coefficients and (95% confidence interval). Standard errors are heteroskedasticity robust (HC0). Pseudo R2—Cragg–Uhler. **p < 0.01, *p < 0.05.

a Median light exposure in the morning (8:00–10:00, unit: 500 lx). Individual data from 7/14 days of actimetry.

b Season of actimetry data collection.

Interestingly, in the model, sex was not significantly associated with having a BDI >10 in this subset of the sample, although the BDI score of women did rank higher than the BDI score of men (Wilcoxon–Mann–Whitney, U = 1,175, *p* < 0.05, n = 115).

## Discussion

Our main results show a relationship between social jetlag (SJL) and depressive symptoms (as subjectively reported in the Beck Depression Inventory, BDI) even in a population experiencing low levels of circadian strain. Furthermore, subjects with clinically significant depressive symptoms are exposed to lower light levels during the day (as objectively measured by actimetry), especially in the morning. The communities we studied have rather similar sociocultural backgrounds.

### Depressive symptoms in Quilombolas

One could expect the depression construct to be different in Quilombos, especially taking into account how geographically isolated some of these communities are. Interestingly, the average BDI-I sum-score and item-total correlation coefficients in our Quilombola sample were similar to the ones reported by Wang et al. (2005) in a population of Chinese immigrants/descendants in the Southeast of Brazil. Items 5 and 7 (“guilty feelings” and “self-dislike”) had item-total correlation coefficients higher than 0.5 in both samples.

Our results of item-total correlations are also somewhat similar to the ones reported in psychiatric patients (Beck and Steer, 1984). The main differences seem to be the low item-total correlation of “suicidal thoughts” in Quilombos and a high coefficient for “health/somatic worries.” Similarly, to what we found in our study, “weight loss” had already shown low item-total correlation both in psychiatric and non-clinical samples (Beck and Steer, 1984; Wang et al., 2005). Additionally, “loss of sexual interest” and “suicidal thoughts,” which had weak item-total correlations in Quilombos, were also reported to have weak item-total correlation and the lowest endorsement rate, respectively, in the BDI-II (Wang et al., 2013).

### Depression and circadian strain

The clash between social schedules and circadian clocks may result in significant disruption of the temporal order of the organisms. A number of metrics can approximate this strain on the circadian system (i.e., *circadian strain*). Here, we used SJL, inter-daily stability (IS) and intra-daily variability (IV). Depressive symptoms were previously shown to be associated with SJL in different populations (Levandovski et al., 2011; Islam et al., 2020), although no difference was found when comparing patients with major depressive disorder and healthy controls (Knapen et al., 2018). In our non-clinical sample, SJL associates with severity/prevalence of depressive symptoms reported in the BDI. Phase of entrainment (using MCTQ-MSF and/or activity acrophase as phase markers) was either not associated with the outcome, or lost significance in the models when social jetlag was added. As previously proposed (Levandovski et al., 2011), this result suggests that the clash between being a late-type and living in a society pervaded by early social times, rather than simply being a late-type, is associated with poor health outcomes. Theories and recent findings from animal models (Landgraf et al., 2016) indicate that disturbances of the biological timing are part of the pathogenesis of mood disorders. Nonetheless, further research is needed to substantiate such causal claims (Bechtel, 2015). In line with Tonon et al. (2017), who proposed actigraphy-derived markers (nocturnal activity) to differentiate subtypes of depression, one could conjecture that circadian strain, either as a risk factor or underlying mechanism, acts specifically on different symptoms of depression. Considering the well-established variability in symptoms of depressed patients, that could be another reason why some studies show significant associations of depressive symptoms with SJL, while others do not.

The fact that SJL but not activity IS was associated with depressive symptoms illustrates the complexity of studying the temporal organization of organisms. Notably, SJL and estimates of stability derived from actimetry are quite different metrics, even if both aim to assess circadian strain to some degree. Circadian misalignment between distinct rhythmic functions may be differentially associated with health outcomes (Roenneberg and Merrow, 2016); in our study, only diurnal rhythms of behavior were assessed. Interestingly, in the subsample with 14 days of temperature recordings, the IS of temperature was also significantly lower in the BDI >10 group, supporting the notion of an association between circadian strain and depressive symptoms.

Our actigraphy sample was similar to the larger sample of questionnaires in terms of BDI, age, and sex distribution (Table 1), but comprised of a higher proportion of individuals from communities less urbanized (e.g., Bombas; Supplementary Table S1). This could also be the reason why actimetry-based estimates of circadian strain (i.e., activity IS, activity IV) were not associated with depressive scores in our study.

### Depression and light exposure

It is hypothesized that changes in environmental light lead to alterations in circadian organization, that in turn contribute to changes in mood. Nevertheless, light also affects mood independently of the clock function (LeGates et al., 2012; LeGates et al., 2014). Besides SJL being associated with clinically relevant BDI scores, we found daytime light exposure to be negatively associated with depression scores. Corroborating our findings of pronounced differences from 8:00 am to 10:00 am, morning exposure to natural light was shown to reduce depression symptoms in seasonal affective disorder (Wirz-Justice et al., 1996) and length of hospitalization in bipolar and depressed patients (Beauchemin and Hays, 1996; Benedetti et al., 2001).

Light exposure in the evening was low in rural Quilombos and did not associate with depressive symptoms. Light may affect mood not only in a circadian-gated manner (e.g., stronger effects at certain times of day) but also by producing changes in the amplitude of the signal and consequently altering the strength of the light/dark cycle as a *zeitgeber*. In our study, it was light during the day and *zeitgeber* strength (measured as the ratio of light exposure in the morning/light exposure in the evening; Supplementary Figure S14) that were negatively associated with BDI scores. These results and those of previous naturalistic studies showing associations either between mood and light exposure during the day (Espiritu et al., 1994; Haynes et al., 2005; Figueiro et al., 2017) or at night (Obayashi et al., 2013; Min and Min, 2018; Obayashi et al., 2018; Paksarian et al., 2020) favor a notion of an integrated light signal (i.e., light history) affecting mood.

Interestingly, in our actigraphy sample, sex was significantly associated with BDI scores in the bivariate analysis, but not in the Poisson regression models, in which light exposure was added as a factor. Women are often responsible for housework and less exposed to light in Quilombos (see Supplementary Figure S13); they also show lower *zeitgeber* strength. These findings suggest that light exposure is an important factor to consider when differences related to sex show in rural communities.

### Study limitations and strengths

The cross-sectional nature of our study does not allow causal inferences. Furthermore, subjects were not medically diagnosed for depression but subjectively queried; with a smaller sample size, results were not as clear regarding circadian strain: no significant association of depressive symptoms was found with activity IS and IV. Yet, our study had a considerably large sample size for the analysis of questionnaires.

We assessed light intensities using actimeters equipped with light sensors worn on the wrist, which most probably do not correspond to those at eye level. Although different actimeters were used in our study, this was unlikely a confounder: 1) we transformed data so that they would correspond (Pilz et al., 2018); 2) sensitivity analyses using data collected with each brand showed similar results. Since there is no well-established method or easy way to develop an algorithm to detect when the light sensor is covered by clothing, there was no way to rule it out as a confounder.

In our study, measurements could not be taken all on the same field trip for every subject. However, less than 14% of the samples had measurements more than 6 months apart from each other (less than 1% for MCTQ-BDI). Considering the previously described stability/reliability of the measurements we used (Beck et al., 1988; Kantermann and Eastman, 2018; Ghotbi et al., 2020), we deem our findings meaningful nonetheless. We also found similar results adjusting our models for season of data collection (March 20–September 22 vs. September 22–March 20).

The BDI was developed in samples of different cultural contexts than Quilombos (Groth-Marnat and Wright, 2016), which required us to adapt the questionnaires in the interviews. Nevertheless, we found Cronbach’s alpha to be well within the range found in other studies described by Beck et al. (1988). This finding suggests that the items of the scale measure a depression construct in Quilombolas; what this construct represents was also characterized in our study (Supplementary Table S2).

Common cause frameworks have been criticized for considering symptoms interchangeable and uncorrelated (Fried, 2015). Yet, sum-scores should still represent a “general psychophysiological load” even if they consider all symptoms as equivalent and do not account for their interactions (Fried and Nesse, 2015). We used a threshold to classify individuals into groups according to having or not clinically significant symptoms, but reckon how conceptually frail this (or any) categorization may be. According to Beck et al. (1988), BDI cut-off scores appropriateness varies according to the characteristics of the samples. We consider the choice of a cut-off of >10 to be adequate for our sample (non-clinical, rural, socio-culturally homogeneous).

The main strength of our study is its “real-life” nature. Even with the difficulties that come with non-controlled conditions of ethological studies, we adjusted our models for variables that could confound the results (e.g., age, sex, season). We did not account for diet, which may affect rest-activity rhythms.

### Concluding remarks

Our results highlight the potential of treatment strategies aimed at decreasing circadian strain and insufficient light exposure during the day, which are suggested areas of further research in Psychiatry. Longitudinal and intervention studies might reveal if prevention and treatment actions developed in the framework of circadian medicine are feasible and effective in minimizing depressive symptoms. Likewise, prospective data are needed not only to confirm causal associations, but also to clarify under how much/for how long one needs to be under circadian strain (and which types of it) for consequences to show.

## Data Availability

The datasets presented in this article are not readily available because the data in this study cannot at this stage be publicly provided. Participants were not asked for consent to make individual data available. Requests to access the datasets should be directed to labcronoesono@hcpa.edu.br.
