# Supplementary material for: Circadian Strain, Light Exposure, and Depressive Symptoms in Rural Communities of Southern Brazil
Source: Front Netw Physiol. 2022 Jan 26;1:779136. doi: 10.3389/fnetp.2021.779136 (PMC10013026; doi:10.3389/fnetp.2021.779136)
Supplement: Supplementary file 1 [file DataSheet1.pdf]

Supplementary Material

Content:

1. Fig S1: Subjects included in the study
2. Fig S2: Communities in the south of Brazil included in the study
3. Fig S3: Distribution of BDI items scores
4. Fig S4: Social jetlag by BDI, age and sex categories
5. Fig S5: Bland-Altman plots for 7d vs. 14d recordings activity and temperature variables (IS, IV, acrophase, amplitude)
6. Fig S6: Bland-Altman plots for 7d vs. 14d recordings of median light exposure
7. Fig S7: Actimetry mean group profiles of 7 days
8. Fig S8: Actimetry median group profiles of 14 days
9. Fig S9: Median light exposure of 14 days by BDI categories
10. Fig S10: Actimetry-derived parameters: group-comparisons with 7 days recordings
11. Fig S11: Actimetry-derived parameters: group-comparisons with 14 days recordings
12. Fig S12: Median light exposure in the morning by BDI, age and sex categories
13. Fig S13: Median light exposure in men vs. women
14. FigS14: Zeitgeber strength vs. BDI
15. Fig S15: Phase assessments
16. Table S1: Individuals included from each community
17. Table S2: Quilombolas occupations
18. Table S3: BDI items scores in Quilombolas

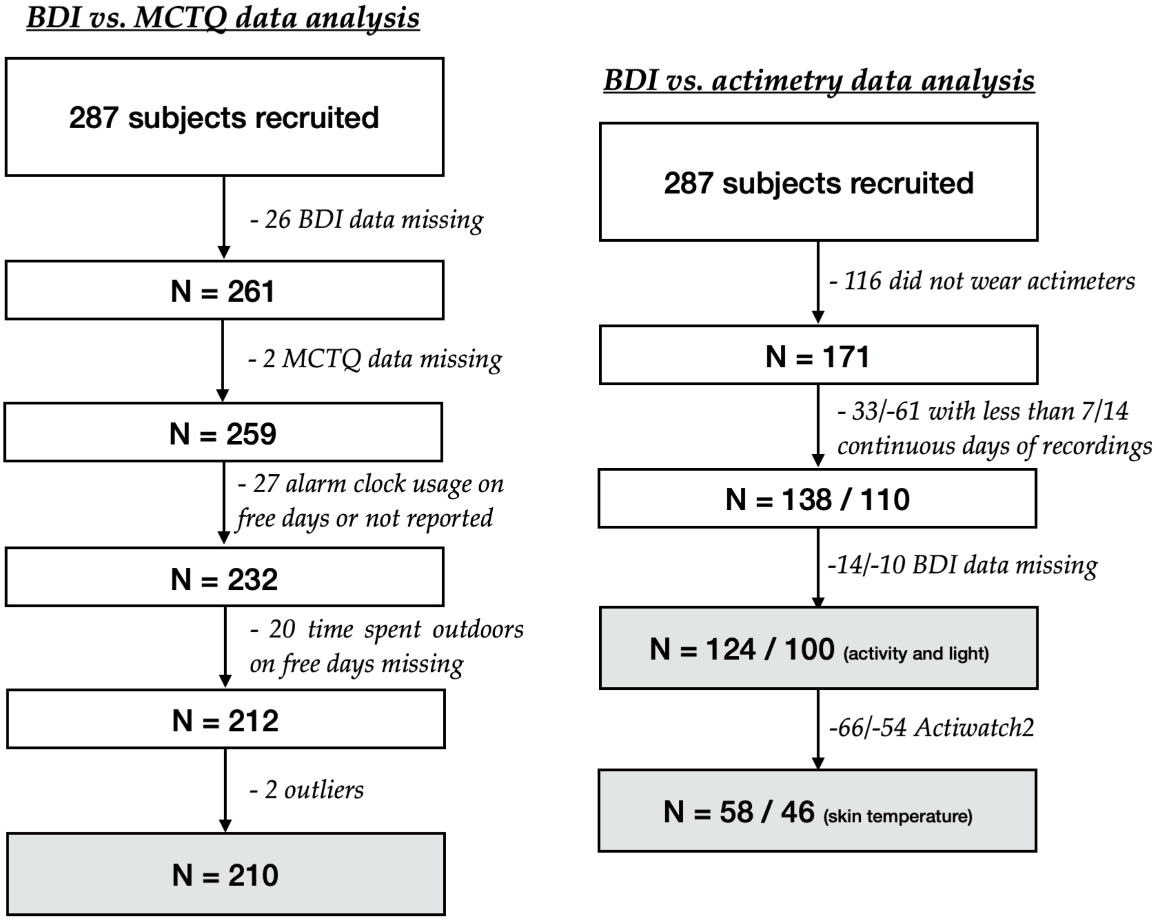

Figure S1. *Subjects included in the study. Grey squares show the final sample size.*

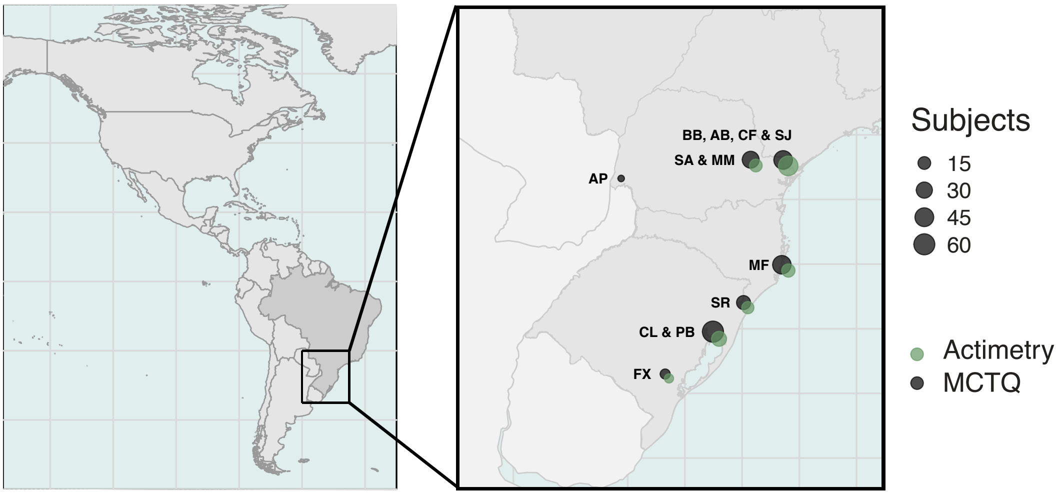

Figure S2. *Communities from the south of Brazil included in the study. The size of the dots corresponds to sample size. AP: Apepu; SA: Serra do Apon; MM: Mamãs; BB: Bombas; AB: Areia Branca; CF: Córrego do Franco; SJ: São João; MF: Morro do Fortunato; SR: São Roque; CL: Cantão das Lombas; PB: Peixoto dos Botinhas; FX: Faxinal.*

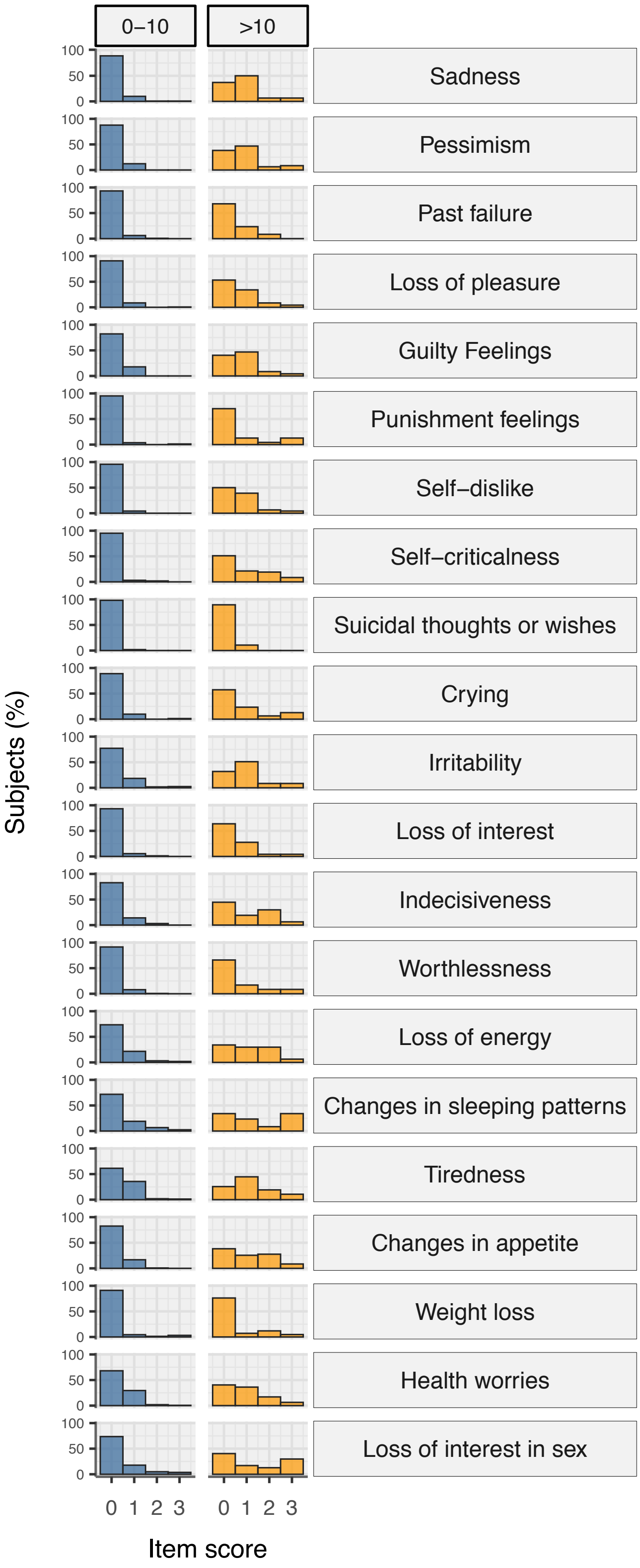

Figure S3. *Distribution of BDI items scores in individuals with BDI sum-score ≤ 10 (n =163) and BDI sum-score >10 (n = 47). Each participant can score from 0 to 3 in each item. N = 210.*

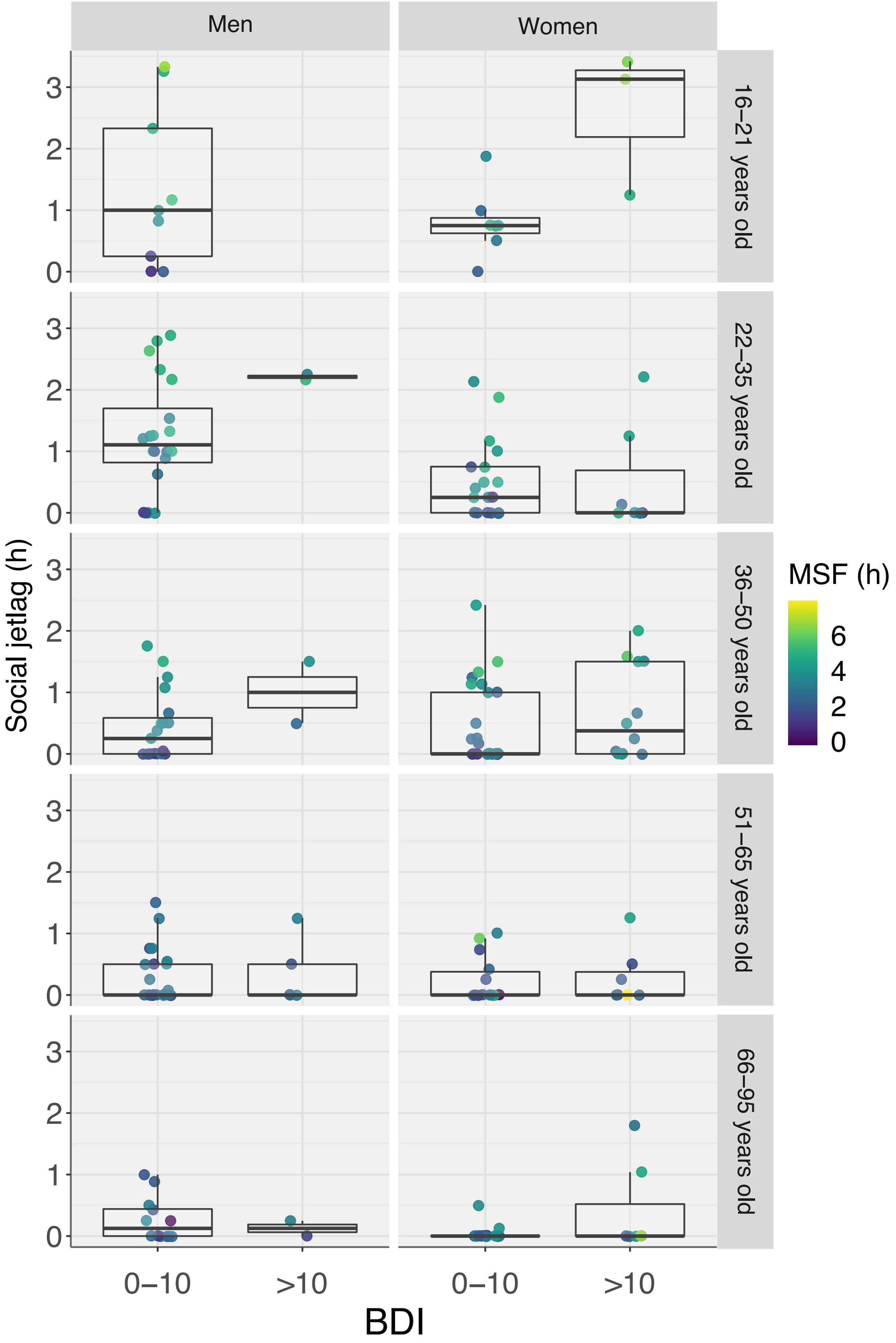

Figure S4. *Social jetlag by BDI, age and sex categories. Dots are color-coded according to midpoint of sleep on free days.*

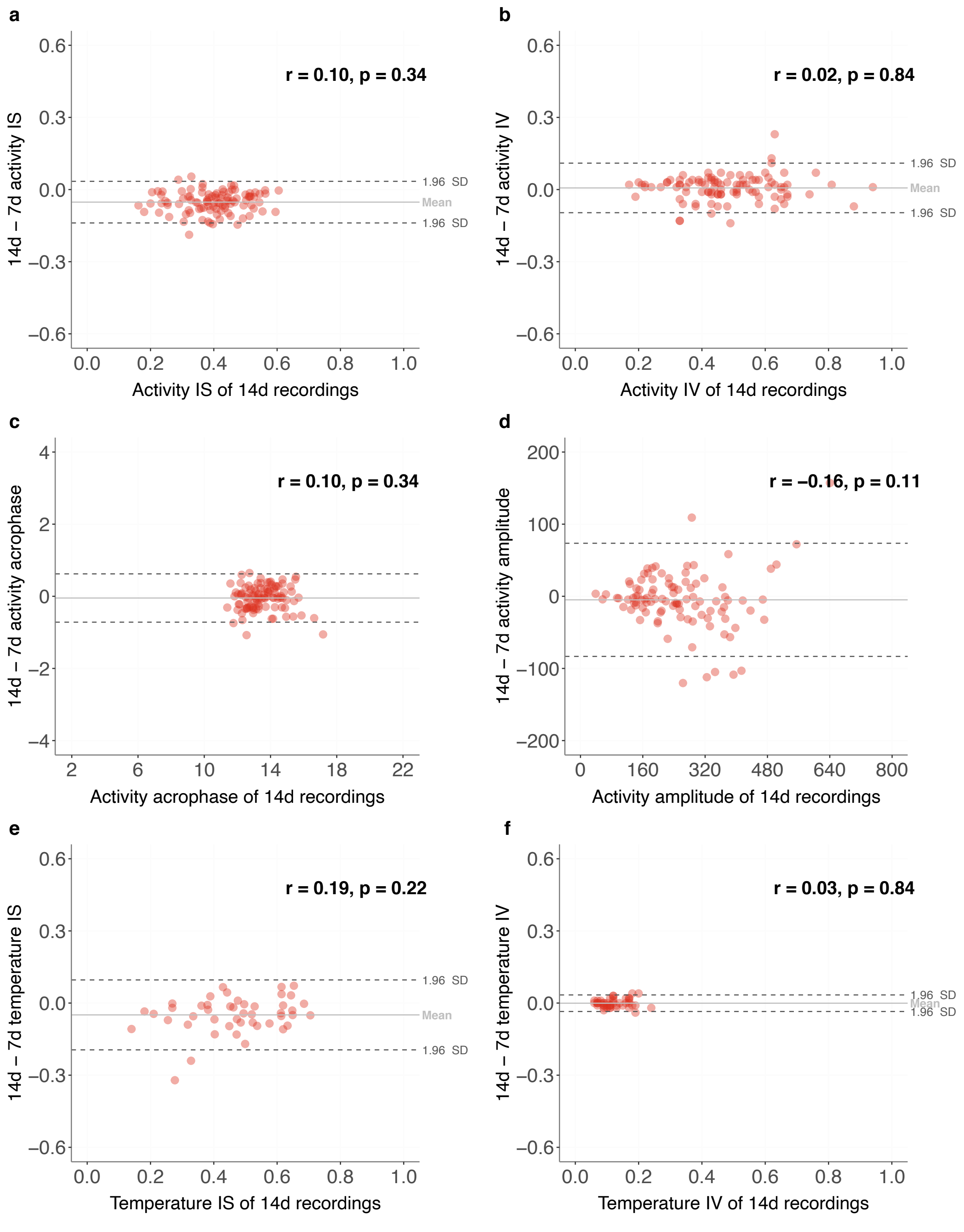

Figure S5. *Bland-Altman plots showing the agreement between estimation of activity and temperature interdaily stability (IS; a, e), intradaily variability (IV; b,f), and activity cosinor-amplitude (c), and cosinor-acrophase (d) using 7 and 14 days of recordings (a-d: n = 100, e-f: n = 46). Mean differences are in general very close to ideal 0 and no remarkably large limit of agreement or pattern along the horizontal axis can be seen, indicating good agreement. There is no significant correlation (Spearman). Limits of agreement are also rather narrow. n = 100 (activity). n = 46 (temperature).*

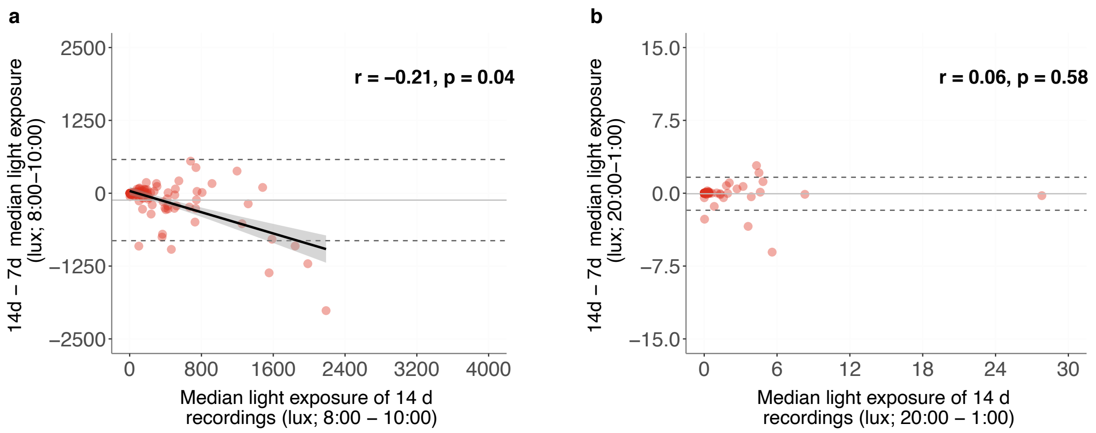

Figure S6. *Bland-Altman plots showing the agreement between median light exposure in the morning (a), and at night (b), using 7 and 14 days of recordings (n =100). Mean differences are very close to ideal 0 and limits of agreement are quite narrow and no pattern is along the x-axis in light at night. Although, Bland-Altman plots of median light exposure in the morning (a) show that measurement bias seems to change in a linear fashion, the correlation between the difference (14d - 7d estimate) and the 14d estimate is not strong. It is a super-estimation of a few of the highest values that seems to make the correlation significant. n =100.*

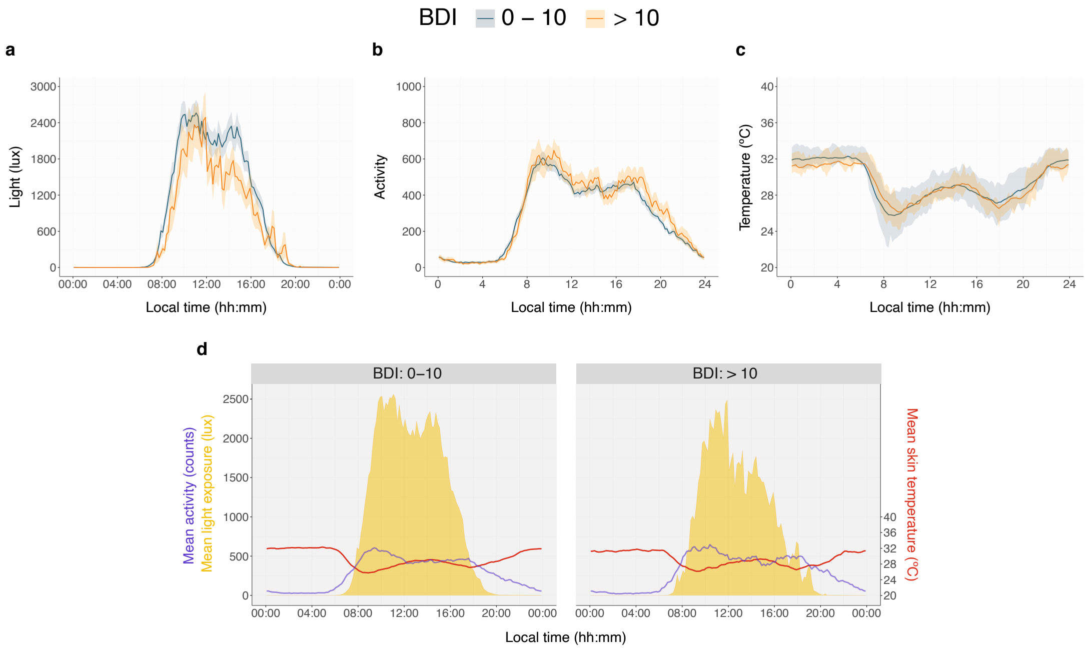

Figure S7. *Light (a), activity (b) and skin temperature (c) group daily profiles of subjects with (orange) and without (blue) clinically significant depressive symptoms. Group profiles were computed using individual* ***mean*** *daily profiles of 7 days. Lines represent* ***mean*** *group daily profiles and the shaded area,* ***standard errors****. The bottom panel shows the same three profiles overlapped. n = 124 (activity/light). n = 58 (temperature).*

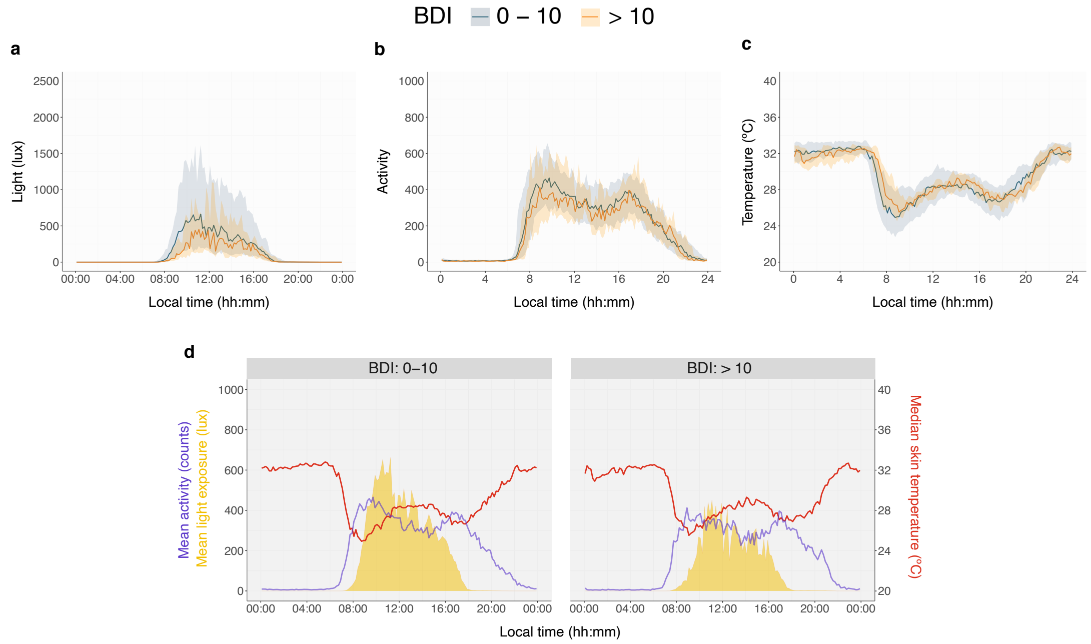

Figure S8. *Light (a), activity (b) and skin temperature (c) group daily profiles of subjects with (orange) and without (blue) clinically significant depressive symptoms. Group profiles were computed using individual* ***median*** *daily profiles of* ***<u>14 days</u>****. Lines represent mean group daily profiles and the shaded area, the* ***interquartile range*** *[Q_1-Q_3]. The bottom panel shows the same three profiles overlaid. n = 100 (activity/light). n = 46 (temperature).*

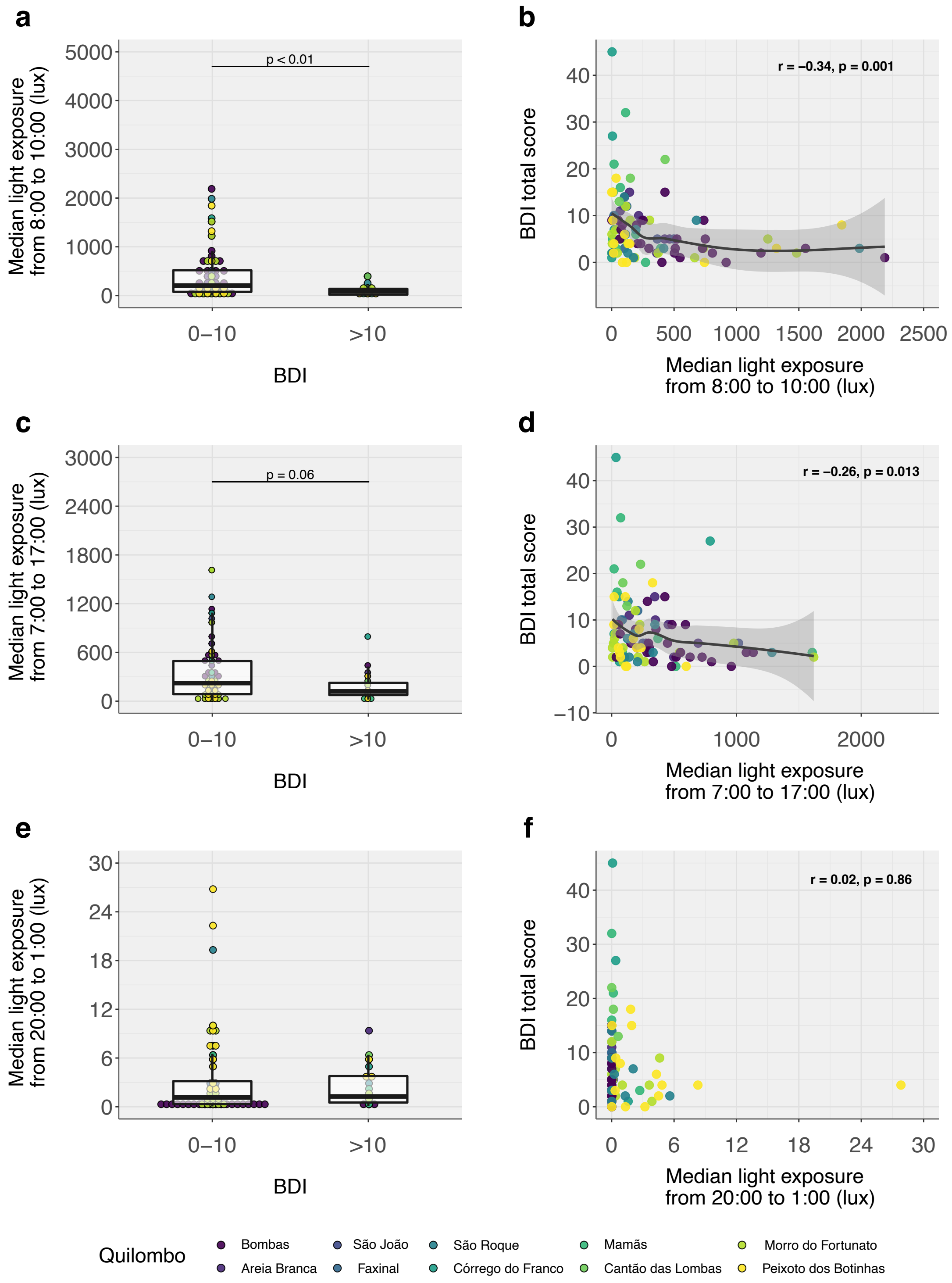

Figure S9. *Light exposure in the morning (from 8:00 to 10:00), during the day (from 7:00 to 17:00) and at night (from 20:00 to 1:00) according to BDI scores (14 days recordings/subject). Subjects with clinically significant depressive symptoms are less exposed to light in the morning (a) and during the day (c), but not at night (e). BDI scores are also negatively correlated to median light exposure in the morning (b) and during the day (d), but not at night (f). Wilcoxon-Mann-Whitney test / Spearman correlation. n = 100.*

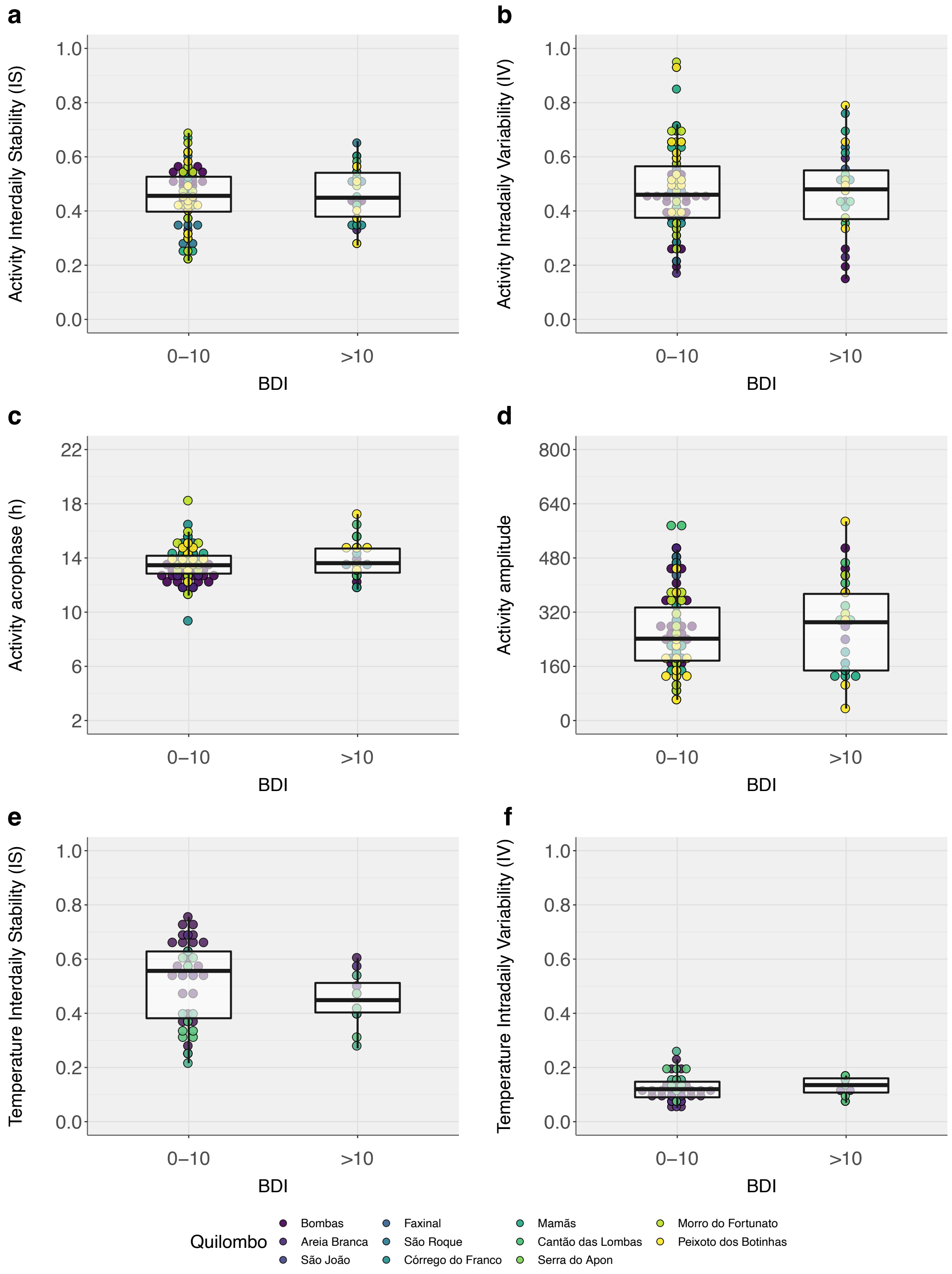

Figure S10. *Comparison of actimetry-derived variables between subjects with and without clinically significant depressive symptoms (7 days recordings/ subject). Wilcoxon-Mann-Whitney test. N = 124/58.*

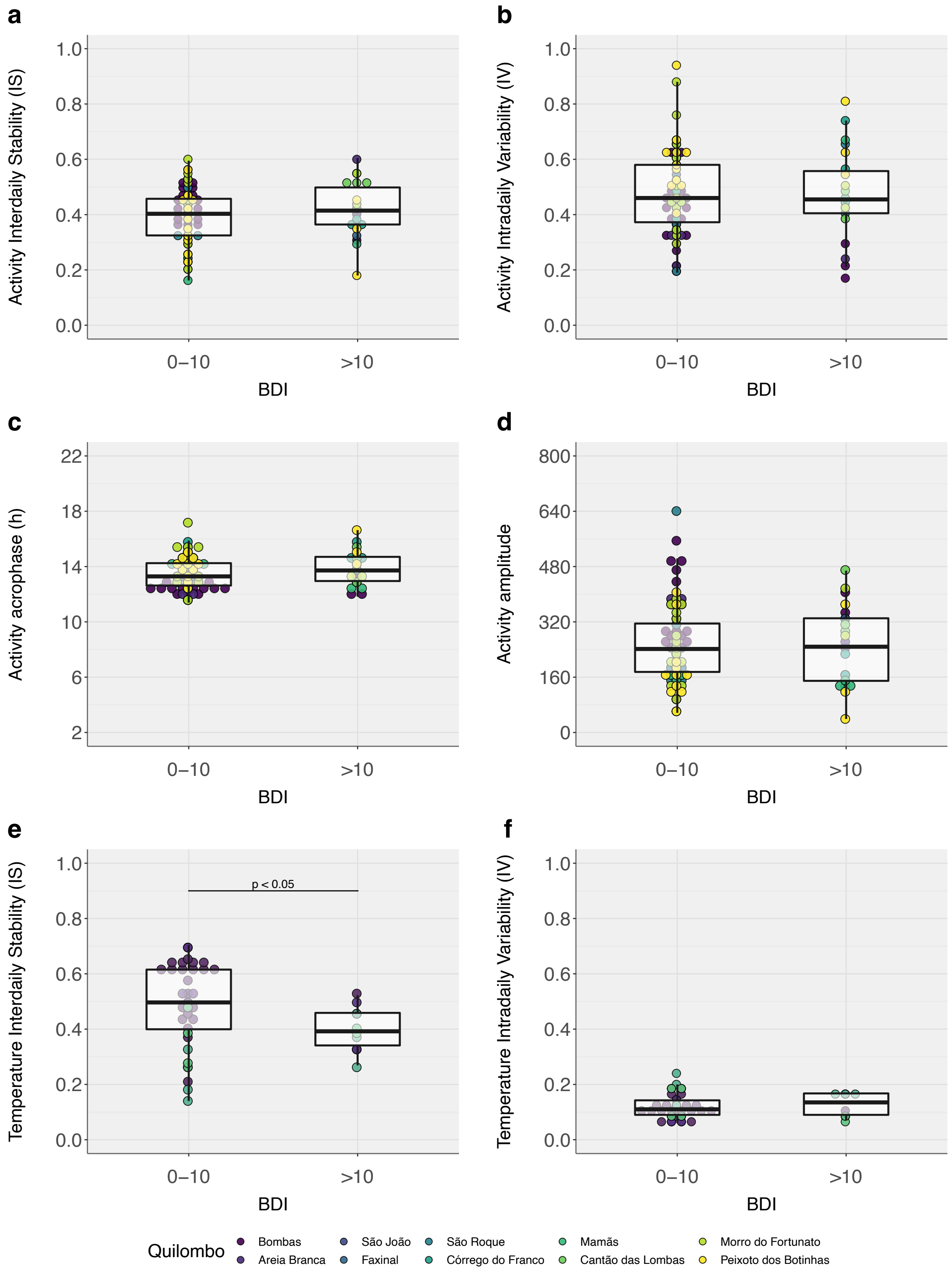

Figure S11. *Comparison of actimetry-derived variables between subjects with and without clinically significant depressive symptoms (14 days recordings/ subject). Wilcoxon-Mann-Whitney test. N = 100/46.*

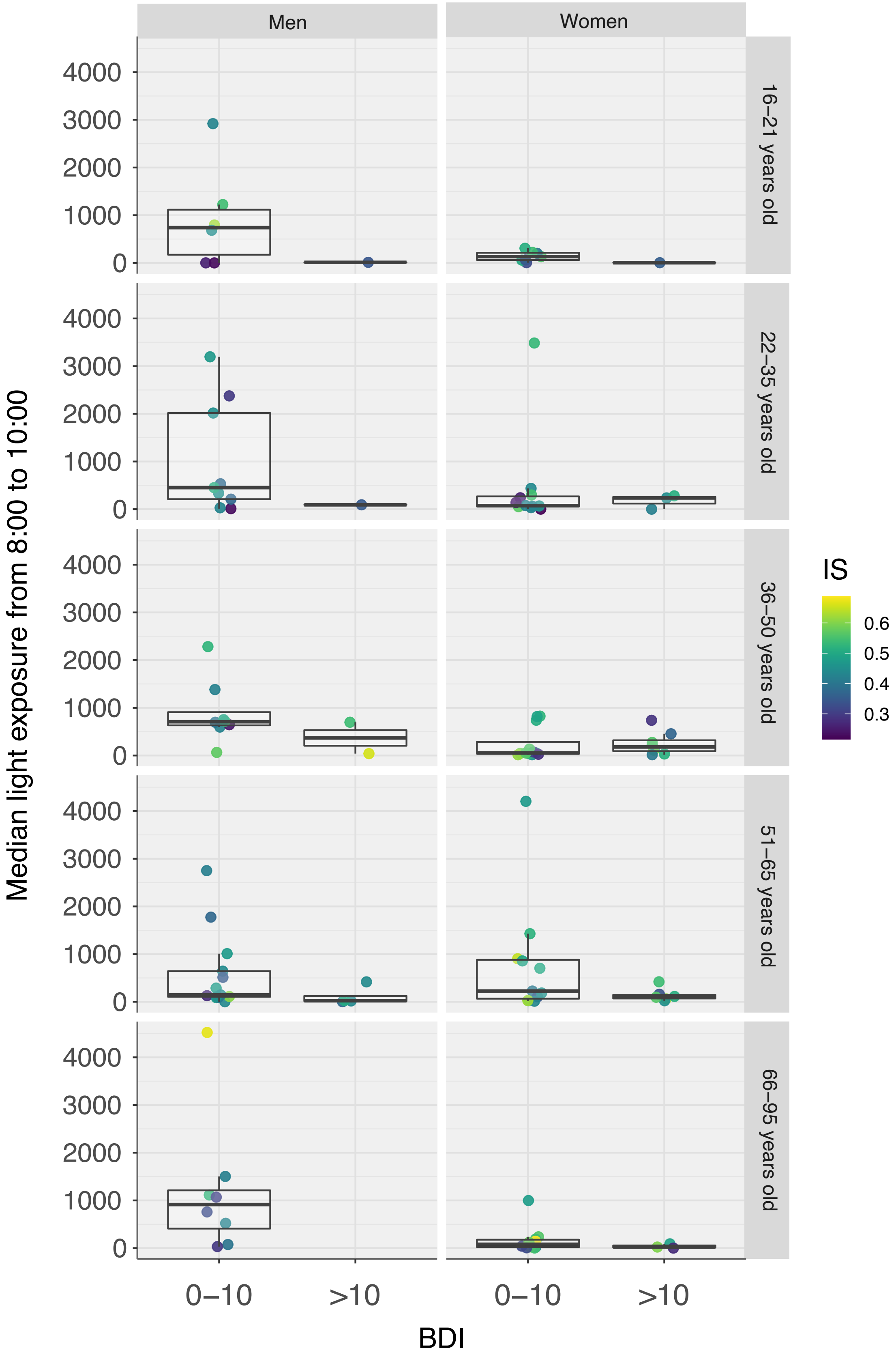

Figure S12. *Median light exposure in the morning (8:00 – 10:00) by BDI, age and sex categories. Dots are color-coded according to interdaily stability (IS).*

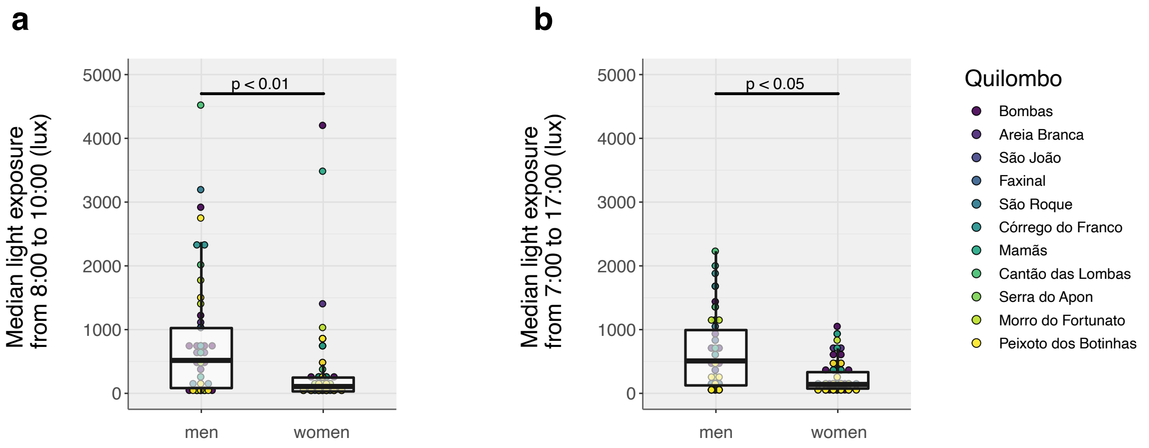

Figure S13. *Median light exposure (lux) in men vs. women: in the morning (a, 8:00 – 10:00) and during the day (b, 7:00 to 17:00). Wilcoxon-Mann-Whitney test.*

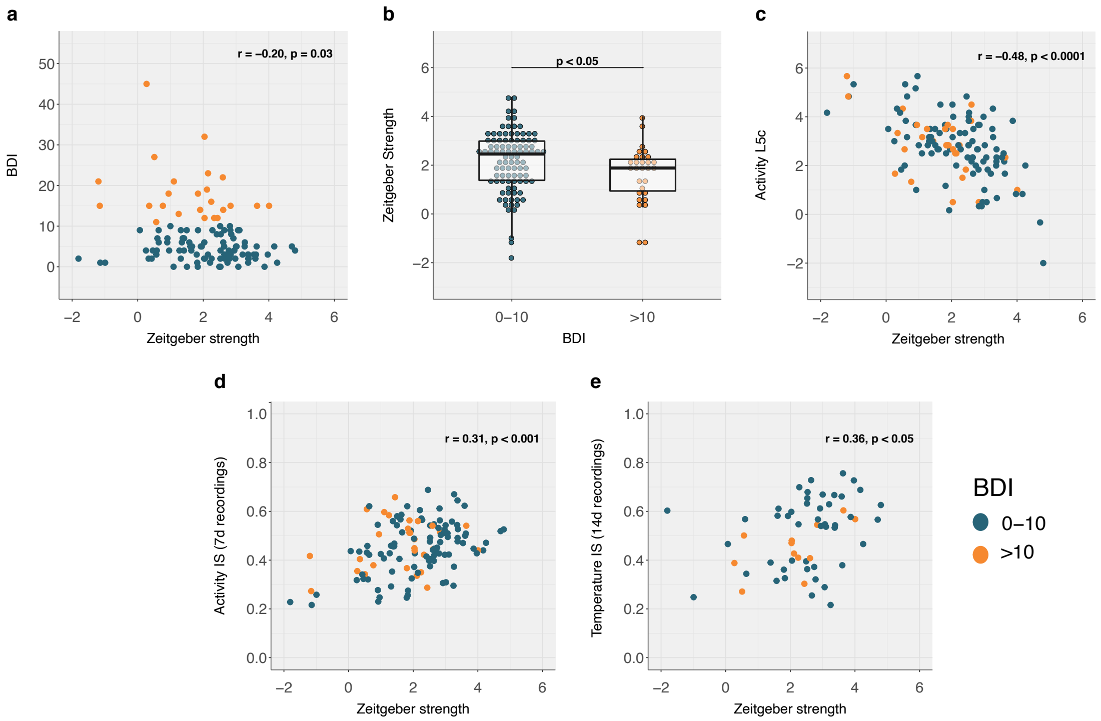

Figure S14. *Zeitgeber strength significantly correlates with BDI scores (a), and is different between BDI categories (b). Zeitgeber strength also correlates with rest phase (L5c; panel c) and IS of activity (d) and temperature (e). Spearman/Wilcoxon-Mann-Whitney test. p < 0.05. Our approximation of* *<u>zeitgeber strength</u>* *was computed as the log10(Median light exposure from 8:00 to 10:00/Mean light exposure from 20:00 to 1:00). Mean was used in the denominator to avoid zeros (*correlation between median and mean light at night is strong – Spearman's rho = 0.80).*

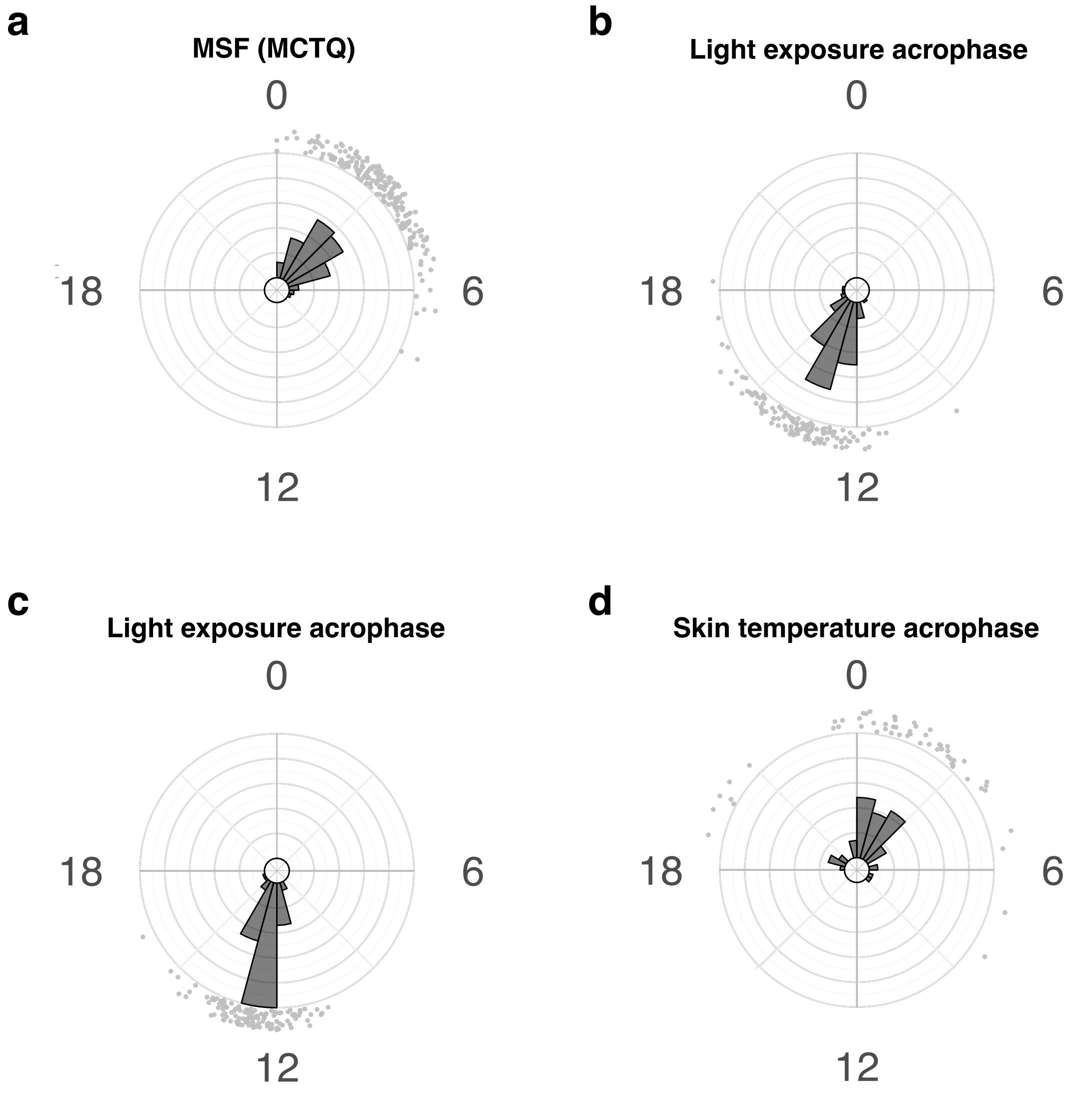

Figure S15. *Distribution of phase variables: (a) midpoint of sleep on free days (MSF) - Munich ChoronoType Questionnaire-derived (N = 210); (b) acrophase of activity (N = 124); (c) acrophase of light (N = 124); (d) acrophase of skin temperature (N = 58). - Cosinor (24h). Circles represent the 24h day and dots along the outermost circumference represent individual times. Each concentric dark-grey circumference represents 10% of the sample.*

TABLE S1: Individuals included from each community in questionnaire and actimetry databases

	MCTQ - sample	Actimetry - sample	
		Activity/ Light	Skin temperature
	n (%)	n (%)	n (%)
Bombas	22 (10)	28 (23)	28 (48)
Areia Branca	8 (4)	10 (8)	10 (17)
São João	5 (2)	3 (2)	-
Apepu	1 (0)	-	-
Faxinal	7 (3)	5 (4)	-
São Roque	20 (10)	13 (10)	-
Córrego do Franco	9 (4)	9 (7)	9 (16)
Mamãs	22 (10)	14 (11)	11 (19)
Serra do Apon	12 (6)	1(1)	-
Cantão das Lombas	23 (11)	7 (6)	-
Morro do Fortunato	43 (20)	17 (14)	-
Peixoto dos Botinhas	38 (18)	17 (14)	-

**colour code approximates a range of urbanization and history of access to electricity (lightest grey = no electricity & not accessible by vehicles, darkest grey = access to electricity for longer & easier access to the city). Percentages might not sum up to 100% due to rounding.*

TABLE S2: Quilombolas occupations - MCTQ and actimetry samples (total %)

	MCTQ (n=210)	Actimetry (n=124)
Farmer	33.3	41.3
Housekeeper	21.4	22.6
Farmer AND housekeeper	2.4	4.0
Retired	13.4	13.7
Elementary occupations* and personal service workers	15.7	8.1
Unemployed	3.3	1.6
Personal care and health workers	0.9	0.8
Teacher	0.5	-
Student	2.4	1.6
Craft and related trade workers	0.9	-
Machine operations (drivers)	3.8	4.0
Clerical support workers	0.5	0.8
Not reported	1.4	1.6

**Cleaners, helpers, construction.*
According to the International Standard Classification of Occupation
Percentages might not sum up to 100 % due to rounding.

TABLE S3: BDI items scores in Quilombolas (N = 194)

**BDI items**	Corrected item-total correlation	Total mean ± SD	Female mean ± SD	Male mean ± SD
1. Sadness	0.59	0.26 ± 0.57	0.36 ± 0.63	0.14 ± 0.46
2. Pessimism	0.53	0.28 ± 0.59	0.35 ± 0.66	0.20 ± 0.48
3. Past failure	0.44	0.14 ± 0.40	0.16 ± 0.44	0.11 ± 0.36
4. Loss of pleasure	0.37	0.22 ± 0.54	0.28 ± 0.61	0.14 ± 0.44
5. Guilty Feelings	0.51	0.26 ± 0.55	0.36 ± 0.62	0.18 ± 0.42
6. Punishment feelings	0.28	0.19 ± 0.63	0.21 ± 0.66	0.16 ± 0.59
7. Self-dislike	0.60	0.19 ± 0.50	0.32 ± 0.62	0.02 ± 0.15
8. Self-criticalness	0.54	0.24 ± 0.65	0.34 ± 0.76	0.11 ± 0.44
9. Suicidal thoughts or wishes	0.15	0.04 ± 0.19	0.04 ± 0.19	0.03 ± 0.18
10. Crying	0.44	0.22 ± 0.60	0.36 ± 0.76	0.05 ± 0.21
11. Irritability	0.40	0.40 ± 0.71	0.55 ± 0.80	0.22 ± 0.51
12. Social withdrawal	0.37	0.17 ± 0.50	0.21 ± 0.55	0.11 ± 0.42
13. Indecisiveness	0.45	0.37 ± 0.70	0.51 ± 0.82	0.20 ± 0.48
14. Worthlessness (body image)	0.43	0.19 ± 0.53	0.27 ± 0.65	0.08 ± 0.27
15. Loss of energy	0.40	0.49 ± 0.78	0.61 ± 0.82	0.34 ± 0.71
16. Changes in sleeping patterns	0.43	0.61 ± 0.98	0.75 ± 1.07	0.44 ± 0.85
17. Tiredness	0.49	0.60 ± 0.76	0.69 ± 0.79	0.48 ± 0.71
18. Loss of appetite	0.37	0.32 ± 0.66	0.37 ± 0.72	0.26 ± 0.54
19. Weight loss	0.09	0.22 ± 0.66	0.15 ± 0.55	0.30 ± 0.76
20. Health worries	0.35	0.45 ± 0.65	0.50 ± 0.69	0.40 ± 0.60
21. Loss of interest in sex	0.29	0.57 ± 0.97	0.83 ± 0.1.15	0.25 ± 0.55
Sum-score mean ± SD		6.44 ± 6.44	8.22 ± 7.19	4.24 ± 4.53
Sum-score median [$Q_1 - Q_3$]		4 [2 – 9]	7 [3 – 12]	3 [1 – 6]
Cronbach's alpha		0.84	0.84	0.78

** Women (n = 107) vs. men (n = 87) sum-score comparison: U = 2828.5, p < 0.001.*
